# Concentration and Potential Non-Carcinogenic and Carcinogenic Health Risk Assessment of Metals in Locally Grown Vegetables

**DOI:** 10.3390/foods14132264

**Published:** 2025-06-26

**Authors:** Muhammad Saleem, Yuqiang Wang, David Pierce, Donald A. Sens, Seema Somji, Scott H. Garrett

**Affiliations:** 1 Department of Pathology, School of Medicine and Health Sciences, University of North Dakota, Grand Forks, ND 58202, USA; muhammad.saleem.1@und.edu (M.S.); donald.sens@und.edu (D.A.S.); seema.somji@und.edu (S.S.); 2 Department of Chemistry, University of North Dakota, Grand Forks, ND 58202, USA; yuqiang.wang@und.edu (Y.W.); david.pierce@und.edu (D.P.)

**Keywords:** vegetables, heavy metals, estimated daily intake, non-carcinogenic risk assessment, carcinogenic risk assessment

## Abstract

Heavy metal contamination in food has become a significant global food safety concern. This study assessed the concentrations of As, Ca, Cd, Co, Cr, Cu, Fe, Hg, Mn, K, Mg, Na, Ni, Se, Pb, and Zn in 13 locally grown vegetables using microwave-assisted acid digestion and ICP-MS. The potential human health risks associated with their consumption were also evaluated. Vegetable samples were collected from the local farmer’s market in Grand Forks, North Dakota. The mean levels (μg/g) of Na, Mg, K, Ca, Fe, Se, Mn, Cu, Zn, Co, Hg, Cr, Ni, As, Cd, and Pb were 1001, 2935, 30474, 686.0, 52.90, 0.171, 37.63, 4.936, 21.33, 0.069, 0.0030, 0.049, 0.736, 0.083, 0.298, and 0.019, respectively, having the following decreasing trend: K > Mg > Na > Ca > Fe > Mn > Zn > Cu > Ni > Cd > Se > As > Co > Cr > Pb > Hg. The highest total metals level was found in spinach, with the following decreasing order: spinach > tomato > sugar beet > white eggplant > cucumber ~ kale > green chili > green bean > dill ~ potato > capsicum > onion > corn. Spinach exhibited the highest concentrations of Cd, Cr, Pb, and Hg, which suggests a higher risk of metal exposure from its consumption. Toxic metals except Cd were found to be lower than the maximum allowable concentrations set by international agencies among the analyzed vegetables, while Cd levels were higher than maximum allowable levels in most of the vegetables. Health risks associated with metal intake by vegetable consumption were evaluated in terms of estimated daily intake (EDI), non-carcinogenic risks were evaluated by the target hazard quotient (THQ) and Hazard Index (HI), and carcinogenic risks were evaluated by target cancer risk (TCR). The EDI values of all the metals were found to be below the maximum tolerable daily intake (MTDI). The highest EDI value for Mn, Zn, Hg, Cr, Cd, and Pb was noted in spinach. THQ values for Cd, Co, and As were higher than 1 in most of the vegetable species analyzed, indicating non-carcinogenic health effects to consumers. HI results also posed a non-carcinogenic health risk associated with the intake of these vegetables. Mean TCR values of Cr, Ni, As, and Cd indicated carcinogenic risk for consumers. This study showed that there are potential health risks with consumption of these vegetables. Lastly, regular monitoring of metal levels in vegetables is suggested/recommended to minimize health risks and support pollution control efforts.

## 1. Introduction

Heavy metal pollution significantly impacts environmental quality and food safety and poses serious health risks to consumers [1,2,3]. Food contamination by toxic substances has gained increasing global attention due to its public health implications, and contaminated food consumption represents the non-occupational pathways to heavy metals exposure [4,5,6,7]. Over the past decade, people have been well aware of the health benefits of vegetables. This has led to a rise in vegetable consumption worldwide. However, beyond quantity, the quality and safety of vegetables have become major concerns among consumers [8,9]. One of the most important components of food quality is the toxic metal contamination level in food products [10,11]. Due to a better understanding of the possibility of food chain contamination, international and national food quality regulations have lowered the maximum permissible levels of toxic metals in food products [11,12]. Vegetables and fruits are major components of the human diet, as they are the source of essential nutrients such as calcium (Ca), magnesium (Mg), copper (Cu), iron (Fe), zinc (Zn), antioxidants, and vitamins. Vegetables are an important component of the daily diets of most people around the world [13,14]; thus, toxic metal-contaminated vegetables can cause detrimental effects on human health [15]. Heavy metals such as Cu and Zn are essential trace metals which are involved in various biochemical processes in plants, including photosynthesis, electron transport, and lignin synthesis [16,17]. However, heavy metals such as Hg, Cd, As and Pb are toxic even in low quantities and may cause toxicity leading to cancer and eventual death [8,9,18,19,20].

Toxic metals accumulate in living organisms for long periods due to their persistent and non-biodegradable nature. The primary cause of metal bioaccumulation in plants is soil heavy metal contamination [15,21,22]. Industrialization, urbanization, and modern trends in agricultural activities enhance the chance of farmland being polluted. Heavy metal accumulation in different tissues depends upon different factors like temperature, moisture, organic compounds, pH, the availability of nutritional components, and plant types [23,24,25]. Heavy metals enter the environment from natural sources through weathering, erosion, and geological processes and from anthropogenic sources such as industrial activities, urban runoffs, agriculture activities, vehicular emissions, the incineration of solid urban waste, etc. [4,5,26,27,28,29,30,31,32,33,34,35,36,37]. Heavy metals contaminate vegetables through accumulation from water, soil, and polluted air [1,38,39,40]. There are three routes of heavy metal exposure in humans: ingestion, dermal, and inhalation. Approximately 90% of human heavy metal exposure occurs through ingestion, most significantly from the consumption of contaminated fruits and vegetables, while the rest of it is from inhalation and dermal routes [21]. Since food is an important source of nutrients and necessary elements, it is significant to measure the risks and benefits of eating different foods. Therefore, the estimation of heavy metal levels in vegetables is used to measure the possible health risk to the consumer [41].

Heavy metal contamination in vegetables was conducted globally, and a similar type of research was also conducted in the USA [42,43]; our study uniquely focuses on vegetables produced locally in Grand Forks, North Dakota. To the best of our knowledge, no published studies have reported heavy metal concentrations in vegetables grown locally in Grand Forks, North Dakota. The United States Food and Drug Administration (FDA) is initiating a pilot program to determine the potential contaminants in school meals (A Research Study of Contaminants in School Meals, RFA—FD-25-06). One of the areas of concern is the possible contamination of heavy metals in foods used in the National School Lunch Program (NSLP). This program requires school lunches to include five meal components: fruit, vegetables, grains, meats or meal alternates, and milk. The USDA sets a basic meal pattern that specifies minimum amounts for each component based on age group. Students must select at least three components, including at least ½ cup of fruit or vegetables, and cannot decline more than two components. The variety that can exist within each of these categories, except for milk, forms a very complex matrix to define the possible presence of heavy metals in a school lunch program. Examples of different components with each category could include the following: for fruit, half of the fruits offered can be 100% juice, but whole and cut-up fruits have more fiber, and a quarter-cup of dried fruit counts as much fiber as ½ cup of fruit; for vegetables, leafy greens count for ½ cup of vegetables; for grains, at least 80% of grains offered weekly must be whole grain-rich, and the remaining grains must be enriched; meat and/or meat alternatives could include meat, poultry, fish, cheese, beans, peanut butter or eggs; for milk, 8 ounces of fluid milk with the minimum fat content. Further increasing the complexity of such an analysis is the farm-to-school program that allows school programs to buy fresh fruits and vegetables from local producers to serve in their lunchrooms. The availability of these produces is seasonal in ND, with corn available in September, which can be served as corn on the cob, canned sweet corn, cornbread, corn tortillas, or corn chips; apples in October; leafy greens such as kale, broccoli, collard greens, lettuce, spinach, cabbage, bok choy, or arugula in November; and potatoes in December. Since these ingredients are seasonal, they add increased complexity to analyzing student lunches, since resources may be acquired from different sources between the fall and summer seasons. The current study analyzed vegetables from a farmer’s market to determine if there was any reason for concern before a very complex and expensive analysis would be initiated to cover the wide spectrum of foods in the school lunch programs. Therefore, the main objectives of the study were (1) to assess the selected heavy metal accumulation in locally grown vegetables in the study area, (2) to compare heavy metal accumulation in different vegetable groups, and (3) to estimate the possible non-carcinogenic and carcinogenic risk assessment caused by the consumption of these vegetables.

## 2. Materials and Methods

### 2.1. Sampling

A total of 82 samples (n) of all 13 locally grown vegetables (Table 1) were collected from the local farmer’s market in Grand Forks, North Dakota. The local farmer’s market known as Town Square Farmer’s Market is held every Saturday from mid-June through September each year. The local farmer’s market provides fresh produce that is grown locally. Grand Forks County, an agricultural county in North Dakota, United States, is located in the Red River Valley region, and the area is approximately 1438 square miles (920,320 acres) with an estimated population of over 70,000 individuals. It is known for its rich and fertile soil. Farming is the major industry in this area, and corn, soybeans, sunflowers, and wheat are among the most common crops [44,45]. For a representative vegetable sample, 2–3 sub-samples (~0.2–0.5 kg) of each vegetable species were collected from different vendors at the farmer’s market and stored in a single bag. Samples were transferred to the lab immediately after collection.

### 2.2. Sample Processing

In the laboratory, samples were washed with deionized water to remove the external adherent materials. Samples were then placed at room temperature in open air for 24 h to dry. The samples were homogenized, ground, and oven-dried at 70–80 °C for constant weight. Dried samples were stored in desiccators until further analysis [46,47]. For metal analysis using inductively coupled plasma mass spectrometry (ICP MS), 0.5 g dried-ground vegetable was weighed in a digestion vessel, and 5 mL concentrated HNO_3_ was added to the sample, contained in the vessel. Samples were digested using a Milestone UltraWAVE digestion unit (Shelton, CT, USA) following EPA method 3051A (Revision 1) and instructions supplied by the manufacturer. The optimal operating conditions were as follows: ramp to 175 °C for 5.5 min, then hold at 175 °C for 4.5 min and N_2_ pressure at 35 bar. After digestion, a clear solution was obtained. The solution was transferred to tubes with plug seal caps, and a final volume of 50 mL was constituted with deionized water. Blanks were also prepared in each batch of samples [48,49].

### 2.3. Quantification of Heavy Metals

The quantitative determination of Na, Mg, K, Ca, Fe, Se, Mn, Cu, Zn, Co, Cr, Ni, As, Cd, and Pb in vegetable samples was performed using a Thermo Scientific iCAP Qc ICP MS in kinetic energy discrimination mode (KED). All operating parameters were in accordance with EPA method 200.8 (Revision 5.4) and the manufacturer’s instructions to meet calibration and analysis requirements. The optimal operating conditions were as follows: RF power of 1550 W, plasma gas flow rate of 14.0 L/min, auxiliary gas flow rate of 0.8 L/min, carrier gas flow rate of 0.8 L/min, nebulizer gas flow of 1.06 L/min, make up gas flow of 0.25 L/min, dwell time of 0.05 sec, spray chamber temperature of 2.7 °C, sampler/skimmer cone of nickel. Sc, Rh and Bi were used as internal standards. Mercury was analyzed by direct determination of the homogenized, ground, and oven-dried solid samples using a Milestone DMA-80 Tri Cell direct mercury analyzer (Shelton, CT, USA). All operating parameters were in accord with EPA method 7473 (Feb. 2007 Revision D) and the manufacturer’s instructions to meet calibration and analysis requirements. The optimal operating conditions were as follows: oxygen carrier gas at 60 psig, dry 70 s at 250 °C, decompose 180 s at 250 °C, catalyst 565 °C, cells 120 °C, purge time 60 s, amalgamator heater time 12 s, recording time 30 s. Liquid calibration standards and solid samples (including standard reference materials) were weighed to 0.1 mg in quartz sample boats before placing them in the autosampler spindle. Calibration acceptance required a minimum three-point curve and regression coefficient ≥ 0.995 based on either linear or quadratic fit. All measurements were done in triplicate.

### 2.4. Quality Control and Quality Assurance

To obtain reliable data, quality control (QC) and quality assurance (QA) were measured by blanks, duplicate samples, and standard reference material (NIST 1567b) in vegetable analyses. One SRM sample, one reagent blank, and one blank spike were included in each analytical batch. The relative percentage difference (RPD, %) of heavy metal levels in the duplicate samples was less than 20%. The recoveries of most of the elements in the reference material were greater than 85% for SRM 1567b. The details of LOD, LOQ, methods blanks, blank spike recovery, and RPD for each metal are given in Supplementary Table S1. All reagents (acids, stock solutions, and multi-element solutions) used in this study were of an analytical grade. The glassware was soaked with a 10% HNO_3_ solution overnight, then washed with deionized distilled water and dried prior to use in this study to reduce cross-contamination [45,50].

### 2.5. Health Risk Assessment of Vegetable Consumption

Heavy metal contamination in vegetables can pose acute and chronic health effects in humans [47,51]. To assess the health risk of heavy metals (Se, Mn, Cu, Zn, Co, Cr, Ni, As, Cd, Pb and Hg) in humans by ingestion of these vegetables, the estimated daily intake of heavy metals (EDI) was found, and non-carcinogenic and carcinogenic risk assessments were conducted in this study.

#### 2.5.1. Estimated Daily Intake (EDI)

The estimated daily intake (EDI) (mg/kg body weight/day) of a heavy metal was assessed as vegetable consumption per day by a person, the mean level of that metal, and the body weight of a consumer. EDI was calculated by the following equation [38,52,53]:

$$EDI=\frac{C\times DI}{BW}$$

where C: average heavy metal concentration in the vegetable measured in this study (µg/g), DI: vegetable consumption per day (kg/day), and BW: average body weight of the consumer (kg). The average daily food intake and body weight for adults used in this study were 0.34 kg/person/day and 70 kg, respectively [46,54,55].

#### 2.5.2. Non-Carcinogenic and Carcinogenic Risk Assessment

The non-carcinogenic health risks assessment related to vegetable intake was determined by the target hazard quotient (THQ) and Hazard Index (HI). Target hazard quotient (THQ) is the non-carcinogenic risk assessment of a heavy metal for its long-term exposure from vegetables and fruits; it was calculated as [38,52]

$$THQ=\frac{EDI}{Rf_{D}}$$

The reference dose (RfD) values used for Se, Mn, Cu, Zn, Co, Hg, Cr, Ni, As, Cd, and Pb in this study were taken from ATSDR, USEPA-IRIS and other published articles [56,57,58,59], shown in Supplementary Table S2. If the THQ value > 1, then there is a possibility for a non-carcinogenic effect, and if THQ < 1, it is be considered to be safe for non-carcinogenic effects [60,61].

To measure the overall non-carcinogenic risk to human consumers, the Hazard Index (HI) was calculated. The Hazard Index (HI) can be expressed as the sum of the hazard quotients for all studied metals:

$$HI=\sum_{n=1}^{i}THQ$$

I = 1, 2, 3…, n. There is no risk if the HI < 1, and if the HI ≥ 1, individuals will experience harmful health effects [52,62].

For carcinogenic risk assessment, target cancer risk (TCR) is calculated as [47,63]:

TCR=EDI×CSFo

EDI: estimated daily intake; CSFo: oral cancer slope factor (mg/kg/day)^−1^, and the value for Cr, As, Pb, Cd, and Ni are given in Supplementary Table S2. The acceptable threshold value of the target cancer risk is 1.0 × 10^−4^, whilst the tolerable/permissible cancer risk value is in the range of 1.0 × 10^−6^–1.0 × 10^−4^ [64,65].

## 3. Results and Discussion

### 3.1. Metal Concentrations of Vegetables

Mean metal levels (mg/kg dry weight) in the selected vegetables studied are shown in Table 1 and Table 2 with the following ranges: sodium 4.357–6434, magnesium 1067–13,517, potassium 10,795–69,105, calcium 9.531–3092, iron 13.36–199.3, selenium 0.056–0.481, manganese 5.388–216.6, copper 1.592–8.928, zinc 11.51–78.76, cobalt 0.015–0.218, mercury 0.0008–0.0113, chromium 0.014–0.179, nickel 0.342–2.139, arsenic 0.009–0.436, cadmium 0.006–0.985, lead 0.007–0.081. The highest total metal concentration was found in spinach, and the lowest level was noted in corn, with the following decreasing order: spinach > tomato > sugar beet > white eggplant > cucumber ~ kale > green chili > green bean > dill ~ potato > capsicum > onion > corn. Similarly to our findings, other studies have also reported that spinach contains the highest metal concentrations [66,67]. K, Mg, Na, and Ca macro elements were the most abundant elements in the vegetables studied. Overall, spinach was a prominent source of these macro elements, followed by tomato, sugar beet, and white eggplant, while onion and corn had the lowest levels. The highest levels of K and Mg were noted in spinach, while high Na and Ca levels were observed in sugar beet and kale, respectively, and the lowest levels of K, Ca, and Na were found in corn. Iron, Zn, Cu, Mn, Se, and Co are essential micro elements that play an important role in humans, and the mean metal levels of the vegetables studied showed the following trend: Fe > Mn > Zn > Cu > Se > Co. The highest levels of Fe, Mn, and Zn were noted in spinach, while the highest levels of Cu, Se, and Co were found in tomato, dill, and green chili, respectively. Similarly, the lowest levels of Fe, Mn, and Se were noted in potato, while Zn, Cu, and Co were observed in capsicum, corn, and onion, respectively. The maximum concentrations of Cd, Cr, Pb, and Hg were noted in spinach, while the highest levels of Ni and As were found in green bean and cucumber, respectively. Moreover, the lowest contents of Ni, Cd, Cr, As, and Hg were observed in spinach, corn, potato, green bean, and green chili, respectively. The lowest concentration of Pb was noted in corn, tomato, and green chili. Overall, based on total mean level of metals in studied vegetables, metals showed the following decreasing trend: K > Mg > Na > Ca > Fe > Mn > Zn > Cu > Ni > Cd > Se > As > Co > Cr > Pb >Hg.

**Table 1:** Average concentration of essential macro metals and essential micro metals (μg/g) in vegetables (*n* = 82).

	*n*		Na	Mg	K	Ca	Fe	Zn	Mn	Cu
Potato	8	Mean	44.51	1138	23,880	32.73	13.36	11.73	5.388	5.463
		SD	33.22	311.4	5577	8.880	4.539	2.851	1.389	1.285
Onion	7	Mean	129.6	1067	18,311	321.7	23.67	12.50	9.046	2.854
		SD	48.21	90.66	1983	50.28	6.289	3.278	1.960	1.047
Tomato	7	Mean	273.5	2425	44,547	338.8	45.40	14.38	20.29	8.928
		SD	127.9	382.8	13,050	152.4	12.45	4.116	5.035	2.906
Sugar Beet	7	Mean	6434	2118	34,222	211.9	25.61	13.04	26.05	5.224
		SD	1869	385.5	12,806	98.74	20.28	5.821	10.84	2.495
Green Chili	7	Mean	56.58	1737	25,561	225.0	44.49	11.50	15.19	5.162
		SD	39.27	100.6	4101	57.80	9.534	4.279	3.355	1.198
Dill	5	Mean	252.9	2728	20,363	1619.9	71.78	38.38	87.78	6.313
		SD	104.4	769.2	5691	440.0	23.67	8.728	22.53	1.477
Corn	7	Mean	4.357	1384	10,795	9.531	17.59	17.39	8.441	1.592
		SD	4.294	571.9	4069	7.051	6.724	6.480	4.551	1.167
Spinach	5	Mean	4348	13,517	69,105	1683.9	199.3	78.76	216.6	3.140
		SD	3279	3426	22,705	351.8	41.21	8.618	37.73	0.856
White Eggplant	5	Mean	59.40	2738	39,199	305.0	27.98	15.34	15.46	8.313
		SD	24.65	368.3	9585	61.25	4.872	2.021	1.605	1.472
Kale	5	Mean	1071	2939	29,971	3092	73.17	16.64	45.24	1.838
		SD	658.7	692.7	5908	420.5	12.26	3.024	13.23	0.439
Green Bean	7	Mean	15.75	2240	23,911	427.9	58.89	20.93	16.07	6.199
		SD	5.789	116.0	5305	112.2	8.548	3.499	8.479	1.776
Capsicum	6	Mean	84.96	1431	22,557	184.2	49.08	11.51	13.04	4.435
		SD	43.53	179.5	5699	44.12	10.92	2.831	3.546	1.240
Cucumber	6	Mean	243.8	2695	33,742	465.7	37.28	15.23	10.70	4.706
		SD	63.09	376.5	9934	116.7	7.678	3.209	2.305	0.855

### 3.2. Metal Concentration Comparison Among Different Vegetable Groups

**Figure 1:**
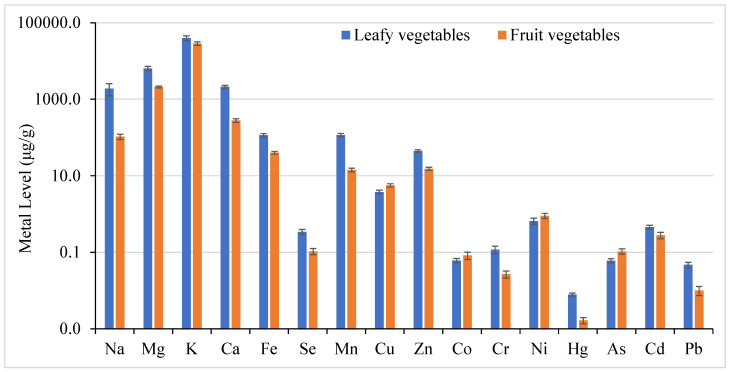
Comparison of metal levels (μg/g ± SE) in leafy vegetables and fruit vegetables.

Based on their characteristics, vegetables can be classified into different groups: leafy vegetables (dill, kale, spinach), fruit vegetables (tomato, green chili, corn, white eggplant, green bean, capsicum, cucumber), root vegetables (sugar beet), bulb vegetables (onion), and stem vegetables (potato). A comparison of metal concentrations was made between fruit vegetables and leafy vegetables (Figure 1). The results showed that leafy vegetables had significantly higher metal concentrations (one-way ANOVA: F ratio > F critical; *p* value < 0.05) than fruit vegetables. Similarly, by comparing the root (sugar beet) vegetable with the stem (potato) vegetable in this study, it was found that most metals showed higher levels in sugar beet samples than potato samples. Various studies have shown that legumes accumulate low amounts, root vegetables moderate amounts, and leafy vegetables high amounts of trace metals [68,69,70]. Leafy vegetables have high heavy metal accumulation, as they have large surface areas and more metals tend to accumulate than in fruits [48,55,71,72,73]. The growth of leafy vegetables is generally faster with higher transpiration rates than non-leafy vegetables, which increases the metal uptake by plant roots in leafy vegetables. Likewise, leafy vegetables are more sensitive to pollutant accumulation in the atmosphere [74,75]. However, heavy metal concentration in different types of vegetables depends on many factors like soil properties, nutrient type, countless environmental and human factors, the nature of the plants, and other soil conditions like pH, organic carbon, etc. [73,76,77,78,79]. Heavy metal accumulation differs among different parts of the plant and varies among cultivars in the same plant species [55,68,69,70,80,81]. The metal concentration in vegetables can vary depending on whether they are consumed raw or cooked. Vegetables are used in both ways. Cooking or peeling vegetables can significantly affect their metal concentration by decreasing or increasing the metal concentration depending upon cooking methods [82,83].

Depending upon their biological role, metals are categorized as essential macro metals (Na, Mg, K, Ca), essential micro metals (Fe, Se, Mn, Cu, Zn, Co, Cr, Ni) and non-essential toxic metals (As, Cd, Hg, Pb) [84,85]. In vegetables, the concentrations of trace metals (As, Cd, Cr, Hg, Pb) that are included in the priority pollutant list by USEPA were compared with the maximum allowable metal concentrations in vegetables set by NHC/FAS [86] and FAO/WHO [87]. Chromium and Hg levels were below the limit set by NHC/FAS: 0.5 mg/kg for Cr and 0.01mg/kg for Hg. Arsenic levels in all vegetables were also noted to be below 0.5 mg/kg, except cucumber, which had a value close to or slightly higher than the limit set by NHC/FAS. Cd and Pb were compared with FAO/WHO standards. Pb levels in leafy vegetables, root/tuber vegetables, and fruit vegetables were lower than the set limits (0.1–0.3 mg/kg) in different vegetables. The maximum levels (mg/kg) of Cd in fruit vegetables, root/tuber vegetables, bulb vegetables, and leafy vegetables are 0.05, 0.1, 0.05, and 0.2, respectively. Cd levels were higher in 100% of tomato, green chili, white eggplant, capsicum, and spinach samples, 86% of onion, sugar beet and potato samples, and 20% of cucumber and green bean samples. Thus, attention and regular monitoring for metals is required in locally grown vegetables. In comparing our data with national research data, there are marked differences where our findings exhibited elevated levels compared to nationally published research data. Lupolt et al. 2021 [42] reported the mean levels of Cr, Pb, Ni, As, and Cd in all vegetables including cucumber, kale, bean, eggplant, tomato, onion, etc. as being 0.042, 0.068, 0.082, 0.005, and 0.042 μg/g, respectively, which are lower than our findings, except for Pb, which is higher than our results. Hadayat et al. 2018 [55] reported the mean concentrations of metals (μg/kg) in five vegetables (lettuce, potato, carrot, tomato, and white onion) as As (7.29), Cd (15.3), Pb (17.9), Cr (46.3), and Ni (58.5), which are also lower than our metal values. Our mean values of Cd, Mn, Cu, Ni, and Zn were also higher than the reported values by Chinnannan et al., 2024 [43]. Leafy green vegetables showed significant levels of Cd and Pb compared to other vegetables as reported by McBride et al., 2014 [88], which is similar to our findings. Thus, attention and regular monitoring for metals is required in locally grown vegetables.

### 3.3. Health Risk Assessment of Metals via Food Consumption

#### 3.3.1. Estimated Daily Intakes (EDIs)

The EDIs of all studied metals except K, Mg, Ca, Na, and Fe were evaluated by the average concentration of each metal in each food, consumption rate, and consumer body weight [89,90] and are shown in Table 3. The EDIs of heavy metals in 13 vegetable samples were as follows: 2.7 × 10^−4^–2.3 × 10^−3^ for Se, 2.6 × 10^−2^–1.1 for Mn, 7.7 × 10^−3^–4.3 × 10^−2^ for Cu, 5.6 × 10^−2^–3.8 × 10^−1^ for Zn, 7.4 × 10^−5^–1.1 × 10^−3^ for Co, 3.7 × 10^−6^–5.5 × 10^−5^ for Hg, 6.8 × 10^−5^–8.7 × 10^−4^ for Cr, 1.7 × 10^−3^–1.0 × 10^−2^ for Ni, 4.1 × 10^−5^–2.1 × 10^−3^ for As, 3.1 × 10^−5^–4.8 × 10^−3^ for Cd, and 3.5 × 10^−5^–3.9 × 10^−4^ for Pb (Table 2). The highest EDI values for Mn, Zn, Hg, Cr, Cd, and Pb were noted in spinach, while the highest EDI values for Se, Cu, Co, Ni, and As were found in dill, tomato, green chili, green bean, and cucumber, respectively. On a mean basis of all vegetables, the EDI showed the following decreasing order: Mn > Zn > Cu > Ni > Cd > Se > As > Co > Cr > Pb > Hg. The EDI values of heavy metals in the studied vegetables were noted to be lower than the maximum tolerable daily intake (MTDI) for individual metals [56], shown in Table 3, indicating that there is no possible human risk associated with daily vegetable consumption from the study area.

#### 3.3.2. Non-Carcinogenic and Carcinogenic Health Risk Assessment

There might be harmful health effects on humans caused by long exposure to toxic metals; thus, carcinogenic and non-carcinogenic parameters are used to assess health risks related to long exposure to toxic metals [47,91]. Non-carcinogenic and carcinogenic health risks were measured by THQ, HI, and TCR. THQ and HI, measuring noncarcinogenic health risk, are acceptable if their values are ≤1 [61,92]. THQ values for Se, Hg, Cr, Ni, and Pb were lower than the critical value (=1.0) in all studied vegetables, indicating no potential harmful effects on consumers. The THQ values were above 1 for Mn in spinach and kale, Zn in spinach, and Cu in white eggplant and tomato. The THQ values for Cd, Co, and As were higher than 1 in most of vegetable species. Cadmium (THQ: 0.03–4.79), Co (THQ: 0.25–3.54), and As (THQ: 0.14–7.05) are the major sources of potential non-carcinogenic risk associated with consumption of the studied vegetables, as shown in Table 4. THQ results differ from EDI results because EDI measures the amount of a substance consumed daily, while THQ assesses the potential non-carcinogenic health risks of long-term exposure by comparing EDI to a reference dose (RfD); usually, doses less than the RfD are unlikely to cause adverse health effects. Thus, THQ includes both EDI and RfD, leading to different results even for the same EDI values. HI exhibits the cumulative effect of heavy metal intake from vegetable consumption. The HI results (Table 4) were higher than 1, ranging from 1.59–18.3, which indicates non-carcinogenic health risks associated with the intake of these vegetables. The highest HI value was noted for spinach, which had higher levels of THQs for Cd, Pb, Cr, Hg, Zn, and Mn.

**Figure 2:**
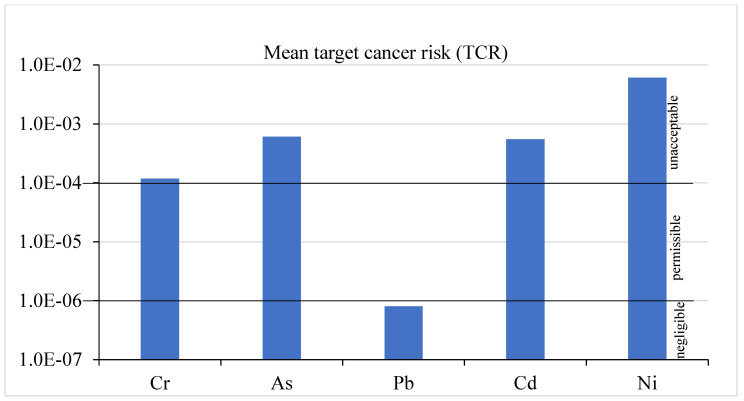
Mean target cancer risk (TCR) of Cr, As, Pb, Cd, and Ni through the consumption of studied vegetables. negligible: TCR < 1.0 × 10^−6^; permissible: 1.0 × 10^−6^ < TCR < 1.0 × 10^−4^; unacceptable: TCR > 1.0 × 10^−4^.

The target cancer risk (TCR) values were calculated for Pb, Cr, Cd, and As (Table 5). TCR < 1.0 × 10^−6^ is considered negligible, while TCR > 1.0 × 10^−4^ is unacceptable, and TCR ranging from 1.0 × 10^−6^–1.0 × 10^−4^ is considered acceptable/permissible [64,65]. The TCR values of Pb for adults were lower than 1.0 × 10^−6^, indicating no carcinogenic risk; the TCR values of As and Cd for onion, tomato, sugar beet, green chili, dill, spinach, white eggplant, kale, and capsicum were higher than 1.0 × 10^−4^; the TCR values for Ni for all vegetables were found to be higher than the permissible limit, which indicates carcinogenic risk for consumers. The TCR values for Cr ranged from permissible to unacceptable risk. Overall, the mean TCR values of Cr, Ni, As, and Cd (Figure 2) were higher than 1.0 × 10^−4^, indicating carcinogenic risk for consumers, indicating that these vegetables may be unsafe for consumption.

## 4. Conclusions

The present study was conducted to assess the metal levels in locally grown vegetables and the potential risk associated with their consumption. The present study results indicate that the highest total metals levels were found in spinach with the following decreasing order: spinach > tomato > sugar beet > white eggplant > cucumber ~ kale > green chili > green bean > dill ~ potato > capsicum > onion > corn. The maximum concentration of toxic metals (Cd, Cr, Pb, and Hg) was noted in spinach, while the highest level of As was found in cucumber. Based on vegetable characteristics, leafy vegetables have higher levels of metal contents than fruit vegetables. Considering the results obtained in terms of the allowable limits of heavy metals in vegetables as set by NHC/FAS and FAO/WHO, Cr, Hg, As, and Pb were below the limit, while Cd levels were higher in 100% of samples of tomato, green chili, white eggplant, capsicum, and spinach. The risk assessment was calculated by EDI, THQ, and TCR. The highest EDI values for Mn, Zn, Hg, Cr, Cd, and Pb were noted in spinach. The THQ values for Cd, Co, and As were higher than 1 in most of the studied vegetable species, indicating potential harmful effects to consumers. HI results indicated non-carcinogenic health risk associated with these vegetables. The highest HI value was noted for spinach, which had higher levels of Cd, Pb, Cr, Hg, Zn, and Mn. TCR results indicated no carcinogenic risk for Pb; TCR values of As and Cd for most of the studied vegetables, while TCR values for Ni for all vegetables were higher than 1.0 × 10^−4^, which indicates carcinogenic risk for consumers. TCR values for Cr ranged from permissible to unacceptable risk. The mean TCR values of Cr, Ni, As, and Cd indicated carcinogenic risk for consumers. Overall, the consumption of vegetables that contain Cd, Ni, As, or Cr can lead to serious health issues such as kidney and liver problems, respiratory disorders, and increased cancer risk for the local community. Thus, there is a need for regular monitoring for toxic metals in locally grown food in the future.

## Figures and Tables

**Table 2:** Average concentration of essential micro metals and non-essential toxic metals (μg/g) in vegetables.

		Se	Co	Cr	Ni	Hg	As	Cd	Pb
Potato	Mean	0.056	0.047	0.014	0.535	0.0017	0.010	0.218	0.009
	SD	0.036	0.024	0.005	0.206	0.0009	0.002	0.087	0.003
Onion	Mean	0.312	0.015	0.032	0.400	0.0010	0.058	0.080	0.014
	SD	0.361	0.005	0.021	0.245	0.0002	0.055	0.033	0.007
Tomato	Mean	0.065	0.070	0.030	0.484	0.0029	0.022	0.369	0.007
	SD	0.046	0.031	0.020	0.223	0.0017	0.011	0.101	0.003
Sugar Beet	Mean	0.082	0.073	0.042	0.349	0.0009	0.090	0.245	0.018
	SD	0.078	0.048	0.043	0.128	0.0002	0.087	0.153	0.016
Green Chili	Mean	0.098	0.218	0.033	0.975	0.0008	0.048	0.657	0.007
	SD	0.062	0.135	0.025	0.284	0.0001	0.025	0.442	0.003
Dill	Mean	0.481	0.030	0.061	1.270	0.0047	0.079	0.286	0.040
	SD	0.172	0.011	0.019	0.685	0.0002	0.024	0.075	0.035
Corn	Mean	0.075	0.020	0.019	0.366	0.0012	0.013	0.006	0.007
	SD	0.045	0.014	0.009	0.172	0.0004	0.009	0.004	0.000
Spinach	Mean	0.225	0.125	0.179	0.342	0.0113	0.064	0.985	0.081
	SD	0.055	0.029	0.063	0.070	0.0035	0.016	0.223	0.013
White Eggplant	Mean	0.070	0.034	0.021	0.922	0.0009	0.190	0.453	0.010
	SD	0.013	0.007	0.013	0.264	0.0002	0.075	0.061	0.013
Kale	Mean	0.327	0.028	0.114	0.365	0.0077	0.039	0.105	0.021
	SD	0.120	0.006	0.088	0.058	0.0009	0.010	0.021	0.003
Green Bean	Mean	0.116	0.067	0.024	2.139	0.0020	0.009	0.014	0.009
	SD	0.055	0.026	0.008	0.774	0.0013	0.001	0.025	0.004
Capsicum	Mean	0.109	0.100	0.028	0.712	0.0017	0.027	0.411	0.008
	SD	0.036	0.090	0.011	0.333	0.0001	0.023	0.304	0.007
Cucumber	Mean	0.208	0.068	0.034	0.713	0.0023	0.436	0.045	0.021
	SD	0.083	0.022	0.013	0.274	0.0013	0.145	0.025	0.013

**Table 3:** Estimated daily intake of heavy metals through the consumption of vegetables in this study.

	Se	Mn	Cu	Zn	Co	Hg	Cr	Ni	As	Cd	Pb
Potato	2.7 × 10^−4^	2.6 × 10^−2^	2.7 × 10^−2^	5.7 × 10^−2^	2.3 × 10^−4^	8.5 × 10^−6^	6.8 × 10^−5^	2.6 × 10^−3^	4.9 × 10^−5^	1.1 × 10^−3^	4.4 × 10^−5^
Onion	1.5 × 10^−3^	4.4 × 10^−2^	1.4 × 10^−2^	6.1 × 10^−2^	7.4 × 10^−5^	4.8 × 10^−6^	1.5 × 10^−4^	1.9 × 10^−3^	2.8 × 10^−4^	3.9 × 10^−4^	6.8 × 10^−5^
Tomato	3.2 × 10^−4^	9.9 × 10^−2^	4.3 × 10^−2^	7.0 × 10^−2^	3.4 × 10^−4^	1.4 × 10^−5^	1.4 × 10^−4^	2.4 × 10^−3^	1.1 × 10^−4^	1.8 × 10^−3^	3.6 × 10^−5^
Sugar Beet	4.0 × 10^−4^	1.3 × 10^−1^	2.5 × 10^−2^	6.3 × 10^−2^	3.6 × 10^−4^	4.4 × 10^−6^	2.1 × 10^−4^	1.7 × 10^−3^	4.4 × 10^−4^	1.2 × 10^−3^	9.0 × 10^−5^
Green Chili	4.7 × 10^−4^	7.4 × 10^−2^	2.5 × 10^−2^	5.6 × 10^−2^	1.1 × 10^−3^	3.7 × 10^−6^	1.6 × 10^−4^	4.7 × 10^−3^	2.3 × 10^−4^	3.2 × 10^−3^	3.5 × 10^−5^
Dill	2.3 × 10^−3^	4.3 × 10^−1^	3.1 × 10^−2^	1.9 × 10^−1^	1.5 × 10^−4^	2.3 × 10^−5^	3.0 × 10^−4^	6.2 × 10^−3^	3.8 × 10^−4^	1.4 × 10^−3^	1.9 × 10^−4^
Corn	3.7 × 10^−4^	4.1 × 10^−2^	7.7 × 10^−3^	8.4 × 10^−2^	9.8 × 10^−5^	5.7 × 10^−6^	9.1 × 10^−5^	1.8 × 10^−3^	6.1 × 10^−5^	3.1 × 10^−5^	3.5 × 10^−5^
Spinach	1.1 × 10^−3^	1.1 × 10^0^	1.5 × 10^−2^	3.8 × 10^−1^	6.1 × 10^−4^	5.5 × 10^−5^	8.7 × 10^−4^	1.7 × 10^−3^	3.1 × 10^−4^	4.8 × 10^−3^	3.9 × 10^−4^
White Eggplant	3.4 × 10^−4^	7.5 × 10^−2^	4.0 × 10^−2^	7.5 × 10^−2^	1.6 × 10^−4^	4.2 × 10^−6^	1.0 × 10^−4^	4.5 × 10^−3^	9.2 × 10^−4^	2.2 × 10^−3^	5.0 × 10^−5^
Kale	1.6 × 10^−3^	2.2 × 10^−1^	8.9 × 10^−3^	8.1 × 10^−2^	1.4 × 10^−4^	3.7 × 10^−5^	5.6 × 10^−4^	1.8 × 10^−3^	1.9 × 10^−4^	5.1 × 10^−4^	1.0 × 10^−4^
Green Bean	5.6 × 10^−4^	7.8 × 10^−2^	3.0 × 10^−2^	1.0 × 10^−1^	3.2 × 10^−4^	9.5 × 10^−6^	1.1 × 10^−4^	1.0 × 10^−2^	4.1 × 10^−5^	6.9 × 10^−5^	4.5 × 10^−5^
Capsicum	5.3 × 10^−4^	6.3 × 10^−2^	2.2 × 10^−2^	5.6 × 10^−2^	4.9 × 10^−4^	8.0 × 10^−6^	1.3 × 10^−4^	3.5 × 10^−3^	1.3 × 10^−4^	2.0 × 10^−3^	3.8 × 10^−5^
Cucumber	1.0 × 10^−3^	5.2 × 10^−2^	2.3 × 10^−2^	7.4 × 10^−2^	3.3 × 10^−4^	1.1 × 10^−5^	1.7 × 10^−4^	3.5 × 10^−3^	2.1 × 10^−3^	2.2 × 10^−4^	1.0 × 10^−4^
Min	2.7 × 10^−4^	2.6 × 10^−2^	7.7 × 10^−3^	5.6 × 10^−2^	7.4 × 10^−5^	3.7 × 10^−6^	6.8 × 10^−5^	1.7 × 10^−3^	4.1 × 10^−5^	3.1 × 10^−5^	3.5 × 10^−5^
Max	2.3 × 10^−3^	1.1 × 10^0^	4.3 × 10^−2^	3.8 × 10^−1^	1.1 × 10^−3^	5.5 × 10^−5^	8.7 × 10^−4^	1.0 × 10^−2^	2.1 × 10^−3^	4.8 × 10^−3^	3.9 × 10^−4^
MTDI *	-		2.5–3	60–65	5.0 × 10^−2^	4.0 × 10^−2^	0.035–0.2	0.1–0.3	1.3 × 10^−1^	0.02–0.07	2.1 × 10^−1^

* MTDI: Maximum tolerable daily intake.

**Table 4:** Target Hazard Quotient (THQ) and Hazard Index (HI) for non-carcinogenic risk assessment associated with vegetable consumption.

	Se	Mn	Cu	Zn	Co	Hg	Cr	Ni	As	Cd	Pb	HI
Potato	0.05	0.19	0.66	0.19	0.77	0.08	0.02	0.13	0.16	1.06	0.01	3.33
Onion	0.30	0.31	0.35	0.20	0.25	0.05	0.05	0.10	0.95	0.39	0.02	2.96
Tomato	0.06	0.70	1.08	0.23	1.14	0.14	0.05	0.12	0.35	1.79	0.01	5.69
Sugar Beet	0.08	0.90	0.63	0.21	1.18	0.04	0.07	0.08	1.46	1.19	0.03	5.88
Green Chili	0.09	0.53	0.63	0.19	3.54	0.04	0.05	0.24	0.77	3.19	0.01	9.27
Dill	0.47	3.05	0.77	0.62	0.48	0.23	0.10	0.31	1.28	1.39	0.05	8.74
Corn	0.07	0.29	0.19	0.28	0.33	0.06	0.03	0.09	0.20	0.03	0.01	1.59
Spinach	0.22	7.51	0.38	1.28	2.03	0.55	0.29	0.08	1.04	4.79	0.11	18.3
White Eggplant	0.07	0.54	1.01	0.25	0.55	0.04	0.03	0.22	3.07	2.20	0.01	8.00
Kale	0.32	1.57	0.22	0.27	0.45	0.37	0.19	0.09	0.63	0.51	0.03	4.65
Green Bean	0.11	0.56	0.75	0.34	1.08	0.10	0.04	0.52	0.14	0.07	0.01	3.71
Capsicum	0.11	0.45	0.54	0.19	1.62	0.08	0.04	0.17	0.43	1.99	0.01	5.64
Cucumber	0.20	0.37	0.57	0.25	1.11	0.11	0.06	0.17	7.05	0.22	0.03	10.1

**Table 5:** Target cancer risk (TCR) of heavy metals for the vegetables studied.

	Cr	As	Pb	Cd	Ni
Potato	3.4 × 10^−5^	7.3 × 10^−5^	3.7 × 10^−7^	4.0 × 10^−4^	4.4 × 10^−3^
Onion	7.7 × 10^−5^	4.3 × 10^−4^	5.8 × 10^−7^	1.5 × 10^−4^	3.3 × 10^−3^
Tomato	7.2 × 10^−5^	1.6 × 10^−4^	3.0 × 10^−7^	6.8 × 10^−4^	4.0 × 10^−3^
Sugar Beet	1.0 × 10^−4^	6.6 × 10^−4^	7.6 × 10^−7^	4.5 × 10^−4^	2.9 × 10^−3^
Green Chili	8.1 × 10^−5^	3.5 × 10^−4^	3.0 × 10^−7^	1.2 × 10^−3^	8.0 × 10^−3^
Dill	1.5 × 10^−4^	5.8 × 10^−4^	1.6 × 10^−6^	5.3 × 10^−4^	1.0 × 10^−2^
Corn	4.5 × 10^−5^	9.2 × 10^−5^	2.9 × 10^−7^	1.2 × 10^−5^	3.0 × 10^−3^
Spinach	4.3 × 10^−4^	4.7 × 10^−4^	3.3 × 10^−6^	1.8 × 10^−3^	2.8 × 10^−3^
White Eggplant	5.0 × 10^−5^	1.4 × 10^−3^	4.3 × 10^−7^	8.4 × 10^−4^	7.6 × 10^−3^
Kale	2.8 × 10^−4^	2.8 × 10^−4^	8.7 × 10^−7^	1.9 × 10^−4^	3.0 × 10^−3^
Green Bean	5.7 × 10^−5^	6.2 × 10^−5^	3.8 × 10^−7^	2.6 × 10^−5^	1.8 × 10^−2^
Capsicum	6.7 × 10^−5^	1.9 × 10^−4^	3.2 × 10^−7^	7.6 × 10^−4^	5.9 × 10^−3^
Cucumber	8.3 × 10^−5^	3.2 × 10^−3^	8.8 × 10^−7^	8.4 × 10^−5^	5.9 × 10^−3^

## Data Availability

Data are contained within article/Supplementary Materials. Further inquiries can be directed to the corresponding author.

## Supplementary Materials

The following supporting information can be downloaded at: https://www.mdpi.com/article/10.3390/foods14132264/s1, Table S1: Limit of Detection (µg/kg), Limit of Quantitation (µg/kg), Method Blank (μg/L), SRM (NIST 1567b) recovery (%) and relative percent difference (RPD, %) of duplicate sample analysis for the selected metals analysis; Table S2: Parameters and variables used in the calculations of THQ and TCR.
