# Supplementary material for: Concentration and Potential Non-Carcinogenic and Carcinogenic Health Risk Assessment of Metals in Locally Grown Vegetables
Source: Foods. 2025 Jun 26;14(13):2264. doi: 10.3390/foods14132264 (PMC12249381; doi:10.3390/foods14132264)
Supplement: Supplementary file 1 [file foods-14-02264-s001.zip › foods-3685553-supplementary.pdf]

foods

MDPI

Concentration and Potential Non-Carcinogenic and Carcinogenic Health Risk Assessment of metals In Locally Grown Vegetables

Muhammad Saleem [1], Yuqiang Wang [2], David Pierce [2], Donald A. Sens [1], Seema Somji [1] and Scott H. Garrett [1,*]

[1] Department of Pathology, School of Medicine and Health Sciences, University of North Dakota, Grand Forks, ND 58202, USA; muhammad.saleem.1@und.edu (M.S.); donald.sens@und.edu (D.A.S.); seema.somji@und.edu (S.S.)
[2] Department of Chemistry, University of North Dakota, Grand Forks, ND 58202, USA; david.pierce@und.edu (D.P.); yuqiang.wang@und.edu (Y.W.)
* Correspondence: scott.garrett@und.edu; Tel.: +1-701-777-2657

 https://doi.org/10.3390/foods14132264

Supplementary Table S1 Limit of Detection (μg/kg), Limit of Quantitation (μg/kg), Method Blank (μg/L), SRM (NIST 1567b) recovery (%) and relative percent difference (RPD, %) of duplicate sample analysis for the selected metals analysis

	LOD	LOQ	Method Blanks	SRM Recovery	PRD
Na	180	560	7.50	121	9
Mg	280	910	1.50	90	10
K	120	390	4.00	95	9
Ca	46	150	3.00	83	4
Fe	250	800	2.80	91	6
Zn	370	1200	2.70	80	11
Mn	3.2	10	0.04	93	9
Cu	9.8	31	0.09	85	9
Cr	1.4	4.3	0.16	-	18
Ni	68	220	0.56	-	18
Se	5.6	18	0.00	84	19
Co	0.63	2	0.005	-	13
As	2.1	6.7	0.003	-	12
Cd	0.79	2.5	0.003	85	7
Pb	1.1	3.5	0.059	98	15

Supplementary Table S2 Parameters and variables used in the calculations of THQ and TCR

	RfD (mg/kg/day)	Reference
Se	0.005	[58,59]
Mn	0.14	[56-59]
Cu	0.04	[56,57]
Zn	0.3	[56-59]
Co	0.0003	[56-58]
Hg	0.0001	[57-59]
Cr	0.003	[56-58]
Ni	0.02	[56,57,59]
As	0.0003	[56-58]
Cd	0.001	[56-58]
Pb	0.0035	[56,57]
	CSFo $(mg/kg/day)^{-1}$	
As	1.5	[56-58]
Pb	0.0085	[56,57]
Cd	0.38	[56,57]
Ni	1.7	[56,57]
Cr	0.5	[56,57]
